# Supplementary material for: Short H2A histone variants are expressed in cancer
Source: Nat Commun. 2021 Jan 20;12:490. doi: 10.1038/s41467-020-20707-x (PMC7817690; doi:10.1038/s41467-020-20707-x)
Supplement: Supplementary file 1 — Supplementary Information [file 41467_2020_20707_MOESM1_ESM.pdf]

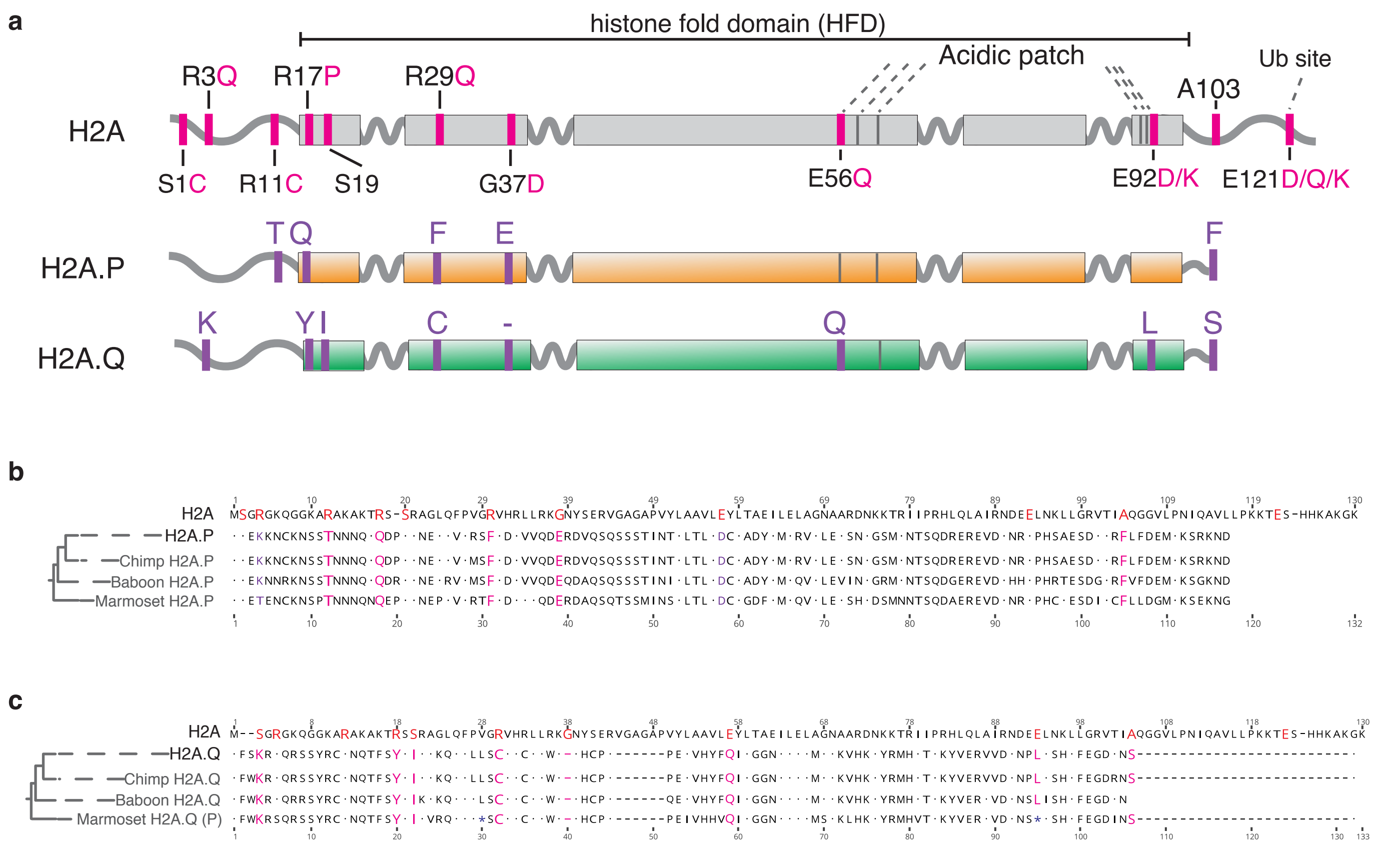

a
histone fold domain (HFD)
Acidic patch
Ub site
H2A
R3Q
R17P
R29Q
A103
S1C
R11C
S19
G37D
E56Q
E92D/K
E121D/Q/K
H2A.P
T Q
F
E
F
H2A.Q
K
Y I
C
-
Q
L
S
b
H2A
H2A.P
Chimp H2A.P
Baboon H2A.P
Marmoset H2A.P
c
H2A
H2A.Q
Chimp H2A.Q
Baboon H2A.Q
Marmoset H2A.Q (P)

Supplementary Figure 1: Other short H2A variants possess oncohistone features that are conserved throughout primates

a. Schematic of common oncomutations found in human core H2A (grey) and their status in H2A.P (orange) and H2A.Q (green).

b. Protein alignment of canonical H2A and H2A.P from Human and representative primates. Substitutions corresponding to oncohistone mutations in H2A (see Fig 1.) are shown in pink.

c. Protein alignment of canonical H2A and H2A.Q from Human and representative primates. Substitutions corresponding to oncohistone mutations in H2A (see Fig 1.) are shown in pink.

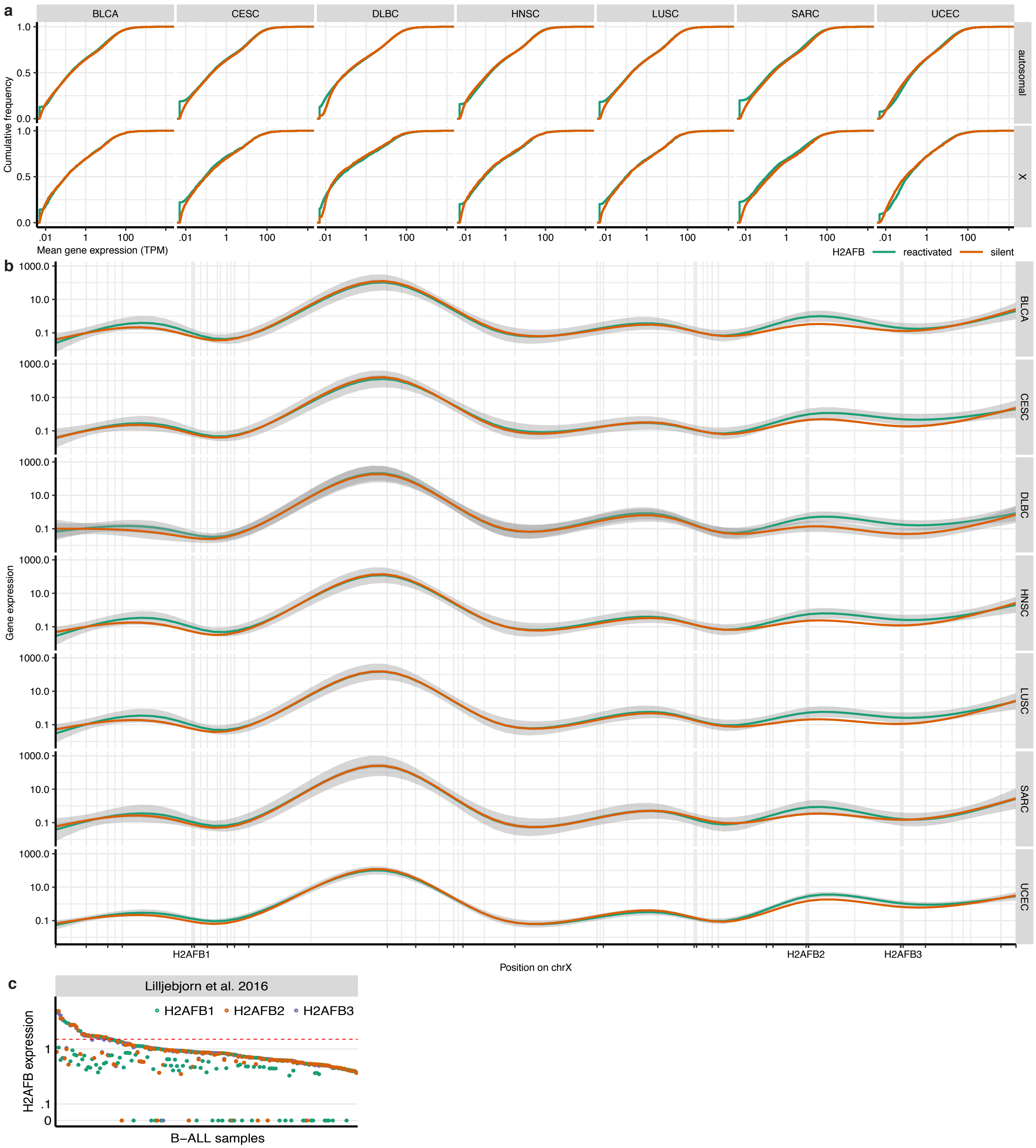
a
BLCA
CESC
DLBC
HNSC
LUSC
SARC
UCEC
autosomal
X
Cumulative frequency
1.0
0.5
0.0
.01
1
100
Mean gene expression (TPM)
H2AFB reactivated silent
b
1000.0
10.0
0.1
Gene expression
H2AFB1
H2AFB2
H2AFB3
Position on chrX
c
Lilljebjorn et al. 2016
H2AFB1 H2AFB2 H2AFB3
H2AFB expression
1
.1
0
B−ALL samples

Supplementary Figure 2:
a. Mean expression of genes on autosomes (above) and the X-chromosome (below) in various cancers (columns) comparing tumors with reactivated (green) or silent (orange) H2A.B, plotted as a cumulative distribution. Only genes with non-zero mean expression (in both reactivated and silent tumors) are shown.
Apart from minor differences in the low range that may be explained by differences in group sizes, there is no substantive difference in the distribution of gene expression magnitude comparing H2A.B-positive vs negative tumors.
b. Expression of genes that lie within positions 154-155Mb of chromosome X (hg19 assembly), which include *H2AFB1/2/3*. Expression of H2A.B reactivated (green) and silent (orange) tumors are compared. Positions of individual genes are indicated as vertical gridlines. Shaded area represents 95% confidence interval from a LOESS fit.
Apart from the specific upregulation of H2A.B in H2A.B-positive samples, there is no evidence of broad regional activation of gene expression that may explain the expression of H2A.B paralogues.
c. As in **Figure 2b**, but for the Lilljebjorn et al. 2016 B-acute lymphoblastic leukemia dataset. The reduced sequencing depth of this dataset (averaging ~23 million mapped reads per sample by TopHat) contributes to greater stochasticity in the estimation of gene expression at the low ranges, and thus while more samples exhibit expression of H2A.B paralogues (~13%), this may in part be explained by underlying stochasticity.

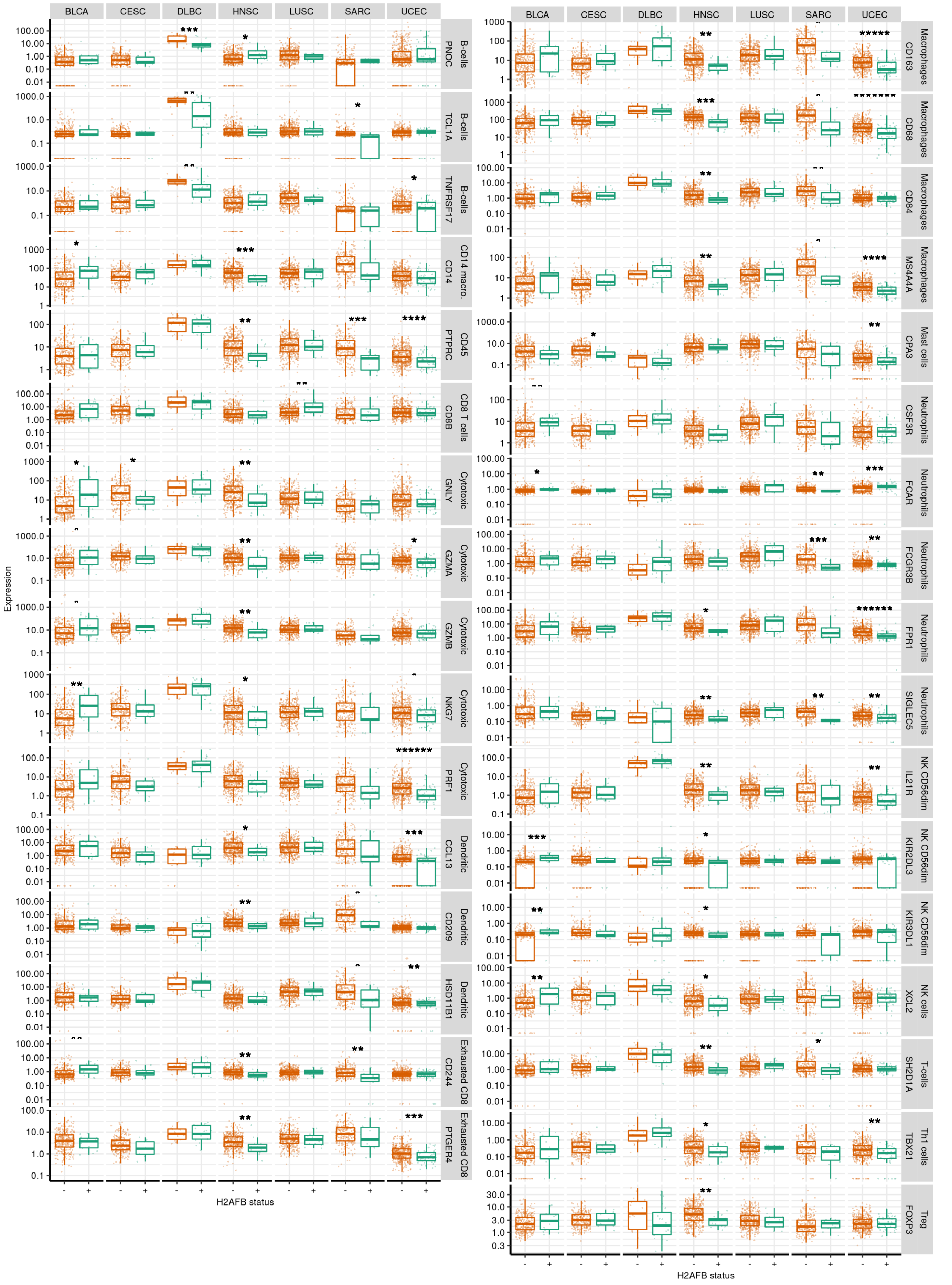

BLCA
CESC
DLBC
HNSC
LUSC
SARC
UCEC
B-cells PNOC
B-cells TCL1A
B-cells TNFRSF17
CD14 macro. CD14
CD45 PTPRC
CD8 T cells CD8B
Cytotoxic GNLY
Cytotoxic GZMA
Cytotoxic GZMB
Cytotoxic NKG7
Cytotoxic PRF1
Dendritic CCL13
Dendritic CD209
Dendritic HSD11B1
Exhausted CD8 CD244
Exhausted CD8 PTGER4
Macrophages CD163
Macrophages CD68
Macrophages CD84
Macrophages MS4A4A
Mast cells CPA3
Neutrophils CSF3R
Neutrophils FCAR
Neutrophils FCGR3B
Neutrophils FPR1
Neutrophils SIGLEC5
NK CD56dim IL21R
NK CD56dim KIR2DL3
NK CD56dim KIR3DL1
NK cells XCL2
T-cells SH2D1A
Th1 cells TBX21
Treg FOXP3
Expression
H2AFB status

Supplementary Figure 3:

a. Box and scatter plots of gene expression (by RNA-seq) of various immune cell markers (rows) in H2A.B positive (green) and negative (orange) tumors in various TCGA cancers (columns). Asterisks show the statistical significance of the difference in marker gene expression by a two-sided Mann-Whitney U test - *: $p < 0.05$; **: $p < 0.01$; ***: $p < 0.001$; ****: $p < 0.0001$ etc. Only immune marker genes where at least one cancer type shows a statistically significant difference in gene expression (at $p < 0.01$) are depicted. Boxplots indicate the 1st quartile, median and 3rd quartile, while the whiskers extend from the box-ends to values no larger / smaller than 1.5 times of the inter-quartile range. All data points are additionally plotted.

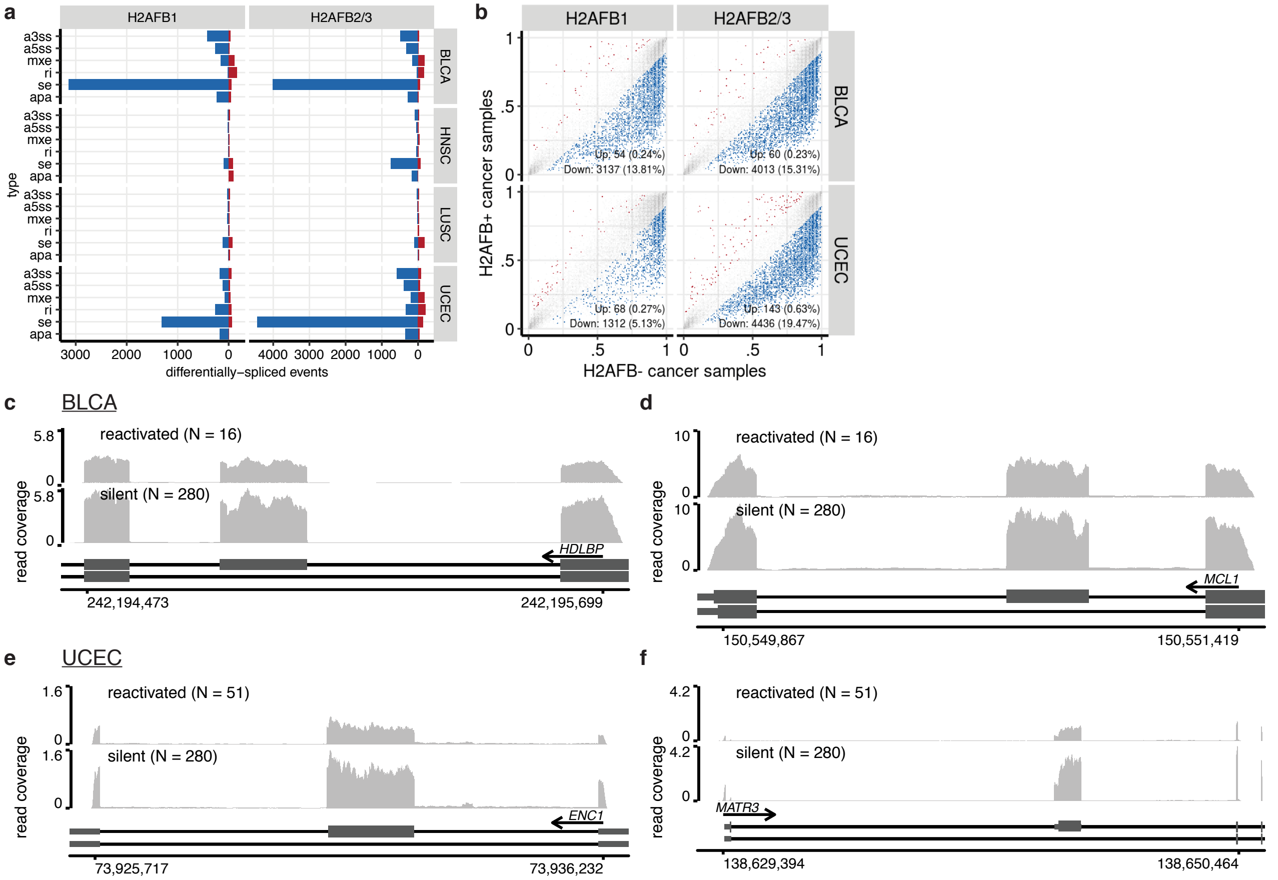
a
H2AFB1
H2AFB2/3
BLCA
HNSC
LUSC
UCEC
type
a3ss
a5ss
mxe
ri
se
apa
differentially−spliced events
b
H2AFB1
H2AFB2/3
Up: 54 (0.24%)
Down: 3137 (13.81%)
Up: 60 (0.23%)
Down: 4013 (15.31%)
Up: 68 (0.27%)
Down: 1312 (5.13%)
Up: 143 (0.63%)
Down: 4436 (19.47%)
H2AFB+ cancer samples
H2AFB- cancer samples
c
BLCA
reactivated (N = 16)
silent (N = 280)
read coverage
HDLBP
242,194,473
242,195,699
d
reactivated (N = 16)
silent (N = 280)
MCL1
150,549,867
150,551,419
e
UCEC
reactivated (N = 51)
silent (N = 280)
ENC1
73,925,717
73,936,232
f
reactivated (N = 51)
silent (N = 280)
MATR3
138,629,394
138,650,464

Supplementary Figure 4:
a. As in **Figure 4a**, but comparing *H2AFB1* (left) or *H2AFB2/3* (right)-positive to negative tumors, for cancer types with at least 5 tumors each expressing either *H2AFB1* or *H2AFB2/3*.
b. As in **Figure 4b**, but for bladder (BLCA) and endometrial (UCEC) cancers, when comparing *H2AFB1* (left) or *H2AFB2/3* (right)-positive to negative tumors.
c. RNA-seq coverage plots at an alternative cassette exon event at *HDLBP*, comparing H2A.B-positive samples with H2A.B-negative samples from bladder cancers.
d. As in **c**, but at *MCL1*
e. As in **c**, but in endometrial cancers at *ENC1*.
f. As in **e**, but at *MATR3*.

Supplementary Table 1. Expression of H2AFB1/2/3 in GTEx normal tissue

Numbers and percentages of samples with H2AFB1, H2AFB2, or H2AFB3 reactivation (expression > 1.5 TPM) from the GTEx dataset of normal tissues, by various tissue types.

GTEx tissue	Number with H2AFB1/2/3 reactivated	Total number	Percentage reactivated
Other	0	7900	0.00
Brain	4	2541	0.16
Colon	1	571	0.18
Thyroid	1	508	0.20
Blood Vessel	3	1320	0.23
Esophagus	6	1364	0.44
Prostate	1	160	0.63
Uterus	1	128	0.78
Spleen	3	202	1.49
Blood	14	929	1.51
Testis	152	252	60.32

Supplementary Table 2: Number of H2AFB1/2/3 reactivated and silent samples in TCGA and B-ALL datasets.

Numbers of cancer samples in TCGA and B-ALL datasets used for gene expression and cancer testes antigen analyses (Fig 3a, b).

Cancer dataset	Reactivated	Silent
BLCA	16	280
CESC	13	231
DLBC	24	17
HNSC	16	442
LUAD	10	476
LUSC	15	409
SARC	10	199
UCEC	51	280
Lilljebjorn et al. 2016	26	69
Liu et al. 2016	11	74
Qian et al. 2017	13	77
Yasuda et al. 2016	4	24
