# Supplementary material for: Short H2A histone variants are expressed in cancer
Source: Nat Commun. 2021 Jan 20;12:490. doi: 10.1038/s41467-020-20707-x (PMC7817690; doi:10.1038/s41467-020-20707-x)
Supplement: Supplementary file 3 — Description of Additional Supplementary Files [file 41467_2020_20707_MOESM3_ESM.pdf]

Description of Additional Supplementary Files

File Name: Supplementary Data 1
Description: Expression of H2AFB1/2/3 in TCGA tumor and normal samples. Numbers and percentages of samples with H2AFB1, H2AFB2, or H2AFB3 reactivated (expression >1.5 TPM) in all cancer types and paired normal tissue from TCGA dataset.

File Name: Supplementary Data 2
Description: Compilation of mutations in selected genes in the TCGA DLBC cohort. Mutations in genes associated with Favorable Prognosis Germinal Center (FP-GC) Subtype (Cluster 4) (as described by Chapuy et al. Nature Medicine 2018) in the TCGA DLBCL cohort

File Name: Supplementary Data 3
Description: Intersection of canonical histone H2A mutations with H2AFB1/2/3-upregulation. Numbers of samples in various cancer types from TCGA dataset with H2AFB1, H2AFB2, or H2AFB3 expression (H2AFB1/2/3 status; reactivated is expression > 1.5 TPM) and mutations in canonical H2A (sporadic or recurrent; see Methods: Somatic mutation analysis).

File Name: Supplementary Data 4
Description: Genes commonly-dysregulated across multiple H2A.B-expressing cancers. Expression of genes that are commonly-dysregulated in multiple cancers (>5). Shown is median expression in H2A.B-expressing or silent tumors and the log2 fold change, where there is a significant difference in expression (Mann-Whitney U test, 2-sided, $p < 0.05$).

File Name: Supplementary Data 5
Description: H2A variant candidate T cell epitopes. Short H2A variant ("H2A variant")-derived peptides ("Peptide sequence") that are predicted to be bound by various common HLA alleles ("HLA allele") and recognized by T-cell receptors, with their calculated NetPanMHCBA4.0 ("NetMHCPan") score (see Methods: Prediction of H2A variant candidate T cell epitopes).

File Name: Supplementary Data 6
Description: . Differential splicing with H2AFB1/2/3-reactivation in various TCGA cancer types. Total number of each splicing event type measured in each TCGA cancer type, with the number of up- or down-regulated events in H2AFB1/2/3-reactivated cancers when compared to H2AFB1/2/3-silent cancers. Abbreviations used for splicing type are as in Fig 2a.
