# Supplementary material for: Short H2A histone variants are expressed in cancer
Source: Nat Commun. 2021 Jan 20;12:490. doi: 10.1038/s41467-020-20707-x (PMC7817690; doi:10.1038/s41467-020-20707-x)
Supplement: Supplementary file 11 — Reporting Summary [file 41467_2020_20707_MOESM11_ESM.pdf]

naturere search

Corresponding author(s): Jay Sarthy and Antoine Molaro

Last updated by author(s): 10/31/2020

Reporting Summary

Nature Research wishes to improve the reproducibility of the work that we publish. This form provides structure for consistency and transparency in reporting. For further information on Nature Research policies, see Authors & Referees and the Editorial Policy Checklist.

Statistics

For all statistical analyses, confirm that the following items are present in the figure legend, table legend, main text, or Methods section.

n/a	Confirmed	
☐	☒	The exact sample size (*n*) for each experimental group/condition, given as a discrete number and unit of measurement
☐	☒	A statement on whether measurements were taken from distinct samples or whether the same sample was measured repeatedly
☐	☒	The statistical test(s) used AND whether they are one- or two-sided<br>*Only common tests should be described solely by name; describe more complex techniques in the Methods section.*
☒	☐	A description of all covariates tested
☒	☐	A description of any assumptions or corrections, such as tests of normality and adjustment for multiple comparisons
☐	☒	A full description of the statistical parameters including central tendency (e.g. means) or other basic estimates (e.g. regression coefficient) AND variation (e.g. standard deviation) or associated estimates of uncertainty (e.g. confidence intervals)
☐	☒	For null hypothesis testing, the test statistic (e.g. *F*, *t*, *r*) with confidence intervals, effect sizes, degrees of freedom and *P* value noted<br>*Give P values as exact values whenever suitable.*
☒	☐	For Bayesian analysis, information on the choice of priors and Markov chain Monte Carlo settings
☒	☐	For hierarchical and complex designs, identification of the appropriate level for tests and full reporting of outcomes
☐	☒	Estimates of effect sizes (e.g. Cohen's *d*, Pearson's *r*), indicating how they were calculated

Our web collection on statistics for biologists contains articles on many of the points above.

Software and code

Policy information about availability of computer code

Data collection	RNA-seq reads from TCGA were downloaded from CGHub. RNA-seq reads from B-ALL samples were obtained from the Japanese Genotype-Phenotype Archive (accession number JGAS00000000047) (Yasuda et al., 2016), and the European Genome-phenome Archive (accession number EGAD00001002112, and EGAD00001002151) (Lilljebjörn et al., 2016 and Qian et al., 2017, respectively), and the Chinese Genotype-phenotype Archive (accession number CGA00000000001) (Liu et al., 2016). RNA-seq quantification of CCLE cell lines were obtained from the Broad Institute CCLE portal (02-Jan-2019 release). TCGA mutation data was obtained through the GenomicDataCommons Bioconductor package.
Data analysis	Reads were processed for gene expression and splice isoform ratio quantification as previously described. Briefly, read alignment and expression estimation were performed with RSEM v1.2.4434, Bowtie v1.0.0445, and TopHat v2.1.1456, using the hg19/GRCh37 assembly of the human genome with a gene annotation that merges the UCSC knownGene gene annotation, Ensembl v71.1 gene annotation, and MISO v2.0 isoform annotation. MISO v2.038 was used to quantify isoform ratios. The trimmed mean of M values (TMM) method, as applied to coding genes, was used to normalize gene expression estimates across all of TCGA, across B-ALL datasets, and across CCLE samples. Data analysis was performed in the R programming environment and relied on Bioconductor, dplyr and ggplot2.

For manuscripts utilizing custom algorithms or software that are central to the research but not yet described in published literature, software must be made available to editors/reviewers. We strongly encourage code deposition in a community repository (e.g. GitHub). See the Nature Research guidelines for submitting code & software for further information.

Data

Policy information about availability of data

All manuscripts must include a data availability statement. This statement should provide the following information, where applicable:

- Accession codes, unique identifiers, or web links for publicly available datasets
- A list of figures that have associated raw data
- A description of any restrictions on data availability

The TCGA data is available through the GDC data portal. RNA-seq reads from B-ALL samples were obtained from the Japanese Genotype-Phenotype Archive

(accession number JGAS00000000047) (Yasuda et al., 2016), and the European Genome-phenome Archive (accession number EGAD00001002112, and EGAD00001002151) (Lilljebjörn et al., 2016 and Qian et al., 2017, respectively), and the Chinese Genotype-phenotype Archive (accession number CGA00000000001) (Liu et al., 2016).

Field-specific reporting

Please select the one below that is the best fit for your research. If you are not sure, read the appropriate sections before making your selection.

☒ Life sciences ☐ Behavioural & social sciences ☐ Ecological, evolutionary & environmental sciences

For a reference copy of the document with all sections, see nature.com/documents/nr-reporting-summary-flat.pdf

Life sciences study design

All studies must disclose on these points even when the disclosure is negative.

Sample size	Sample sizes were determined by the expression status of H2AFB1/2/3 for individual samples within a cancer type: cancer types with fewer than 5 H2AFB1/2/3 expressing samples were not considered for statistical analyses.
Data exclusions	For certain analyses (detailed in the methods) samples with intermediate levels of expression of H2AFB1/2/3 were excluded in the analyses.
Replication	As these analyses use existing data, no replicates were performed. However, the same analyses were performed in various cancers (which are essentially independent), and with either H2AFB1 or H2AFB2/3 for certain cancer types.
Randomization	Randomization is not relevant to this study: samples were segregated by their expression status of H2AFB1/2/3 for comparisons.
Blinding	Blinding is not relevant to this study.

Reporting for specific materials, systems and methods

We require information from authors about some types of materials, experimental systems and methods used in many studies. Here, indicate whether each material, system or method listed is relevant to your study. If you are not sure if a list item applies to your research, read the appropriate section before selecting a response.

Materials & experimental systems

n/a	Involved in the study	
☒	☐	Antibodies
☒	☐	Eukaryotic cell lines
☒	☐	Palaeontology
☒	☐	Animals and other organisms
☒	☐	Human research participants
☒	☐	Clinical data

Methods

n/a	Involved in the study	
☒	☐	ChIP-seq
☒	☐	Flow cytometry
☒	☐	MRI-based neuroimaging
